# iRhom2-mediated proinflammatory signalling regulates heart repair following myocardial infarction

**DOI:** 10.1172/jci.insight.98268

**Published:** 2018-02-08

**Authors:** Damien N. Barnette, Thomas J. Cahill, Mala Gunadasa-Rohling, Carolyn A. Carr, Matthew Freeman, Paul R. Riley

**Affiliations:** 1 Department of Physiology, Anatomy and Genetics, University of Oxford, Oxford, United Kingdom (UK).; 2 Department of Cardiovascular Medicine, University of Oxford, Level 6, West Wing, John Radcliffe Hospital, Oxford, UK.; 3 Sir William Dunn School of Pathology, University of Oxford, Oxford, UK.

**Keywords:** Cardiology, Immunology, Cardiovascular disease, Innate immunity

## Abstract

The role of proinflammation, and specifically TNF-α, on downstream fibrosis and healing after cardiac injury remains unknown. Using iRhom2-deficient mice, which lack myeloid-specific shedding of TNF-α, we reveal increased macrophages (MΦs) that were skewed towards a more proinflammatory (M1) state at day 4, followed by more reparative, antiinflammatory (M2) state at day 7 after myocardial infarction (MI). However, associated functional cytokine expression was significantly reduced in iRhom2-mutant M1 and M2 MΦs, respectively. A dampened proinflammatory signature in iRhom2-deficient mice during the acute phase of injury and subsequent changes in MΦ polarization were associated with reduced phagocytosis and a more sparse distribution within the scar region. This resulted in impaired collagen deposition and fibrosis, and increased left ventricular remodelling and mortality in iRhom2-deficient mice after MI. Our findings reveal a requirement for an iRhom2-mediated proinflammatory response during downstream scarring and fibrosis, which is driven in part by TNF-α signaling. These conclusions challenge the existing model that infarct repair is determined exclusively by antiinflammatory signaling of M2 MΦs, and as such we propose an alternative view of immunomodulation to maintain effective healing after infarction.

## Abstract 2

Optimal scarring and survival after myocardial infarction is dependent upon the initial wave of inflammation after injury.

## Introduction

Myocardial infarction (MI) occurs when a coronary artery becomes obstructed, which leads to an insufficient supply of nutrients and oxygen to myocardium lacking vessel perfusion. Sudden death of cardiomyocytes within the infarcted region activates immune pathways that elicit an acute inflammatory response as a defense mechanism to prevent irreversible damage to the myocardium (1). The current model of innate immunity following MI proposes discrete inflammatory and reparative phases, defined by mutually exclusive populations of immune cells. An early influx of proinflammatory Ly6C^hi^ monocytes (Mos) from bone marrow or splenic reservoirs give rise to classically activated M1 macrophages (MΦs), which promote inflammation, digestion of infarcted tissue, and removal of dying cells and necrotic debris. This is followed by a second influx of antiinflammatory Ly6C^lo^ Mos that are associated with alternatively activated M2 MΦs, which dominate during the resolution of inflammation, propagating repair through collagen deposition to ensure the structural integrity of the left ventricle (2–6).

TNF-α is a proinflammatory cytokine produced predominantly by resident and infiltrating Mos and MΦs immediately following cardiovascular injury (2, 7). TNF-α exerts multiple effects on cardiovascular cell types to regulate processes important for maintaining cell survival, tissue integrity, and function following MI, including ventricular contractility, cardiomyocyte apoptosis, and extracellular matrix formation (8–11). However, the relative significance of these effects on tissue repair and wound healing is contentious, as conflicting studies show TNF-α to be paradoxically cardioprotective and deleterious (7, 10, 12, 13). Determining TNF-α function is challenging, largely owing to its pleiotropic actions and complex regulation through autocrine and paracrine signaling, mediated by functionally distinct type I and II TNF receptors (TNFRI/II). Both ligand and receptors are initially expressed as transmembrane proteins that later undergo proteolytic cleavage by the TNF-α converting enzyme (TACE), which results in the production of their soluble isoforms and subsequent paracrine effects on neighboring cells (14, 15).

The maturation of TACE and its translocation to the cell membrane is regulated by iRhom2, an inactive member of the rhomboid intramembrane family of serine proteases. Although inactive rhomboids lack proteolytic activity, they have been shown to have crucial roles in protein degradation, trafficking regulation, and inflammatory signaling (16). Loss of *Rhbdf2,* which encodes iRhom2, has previously been shown to be devoid of functional TACE in myeloid cells, which cannot be compensated for by the related protease iRhom1 (17, 18). This results in tissue-specific deregulation of downstream secretion and signaling of TACE targets, including TNF-α, through selectively preventing their maturation in immune cells without affecting other cell types.

Using iRhom2-deficient mice, we sought to determine the effects of diminished proinflammation in Mo/MΦ populations following MI. Here, we demonstrate that MΦs require iRhom2 signaling in order to initiate the early inflammatory phase after MI, which unexpectedly altered the late-stage reparative phase of wound healing. iRhom2-deficient MΦs were characterized by significantly reduced M1 and M2 cytokine profiles during the proinflammatory and reparative phases respectively, and increased cell surface TNFR expression. This resulted in impaired cardiac function and defective collagen deposition, which ultimately resulted in a less rigid scar and decreased survival in iRhom2 mutants following MI. Therefore, we implicate a potentially novel, direct role for the iRhom2-mediated acute inflammatory phase in MΦ function, downstream scar integrity, and tissue repair following MI.

## Results

### Loss of iRhom2 alters the inflammatory response after cardiac injury.

**Figure 1:**
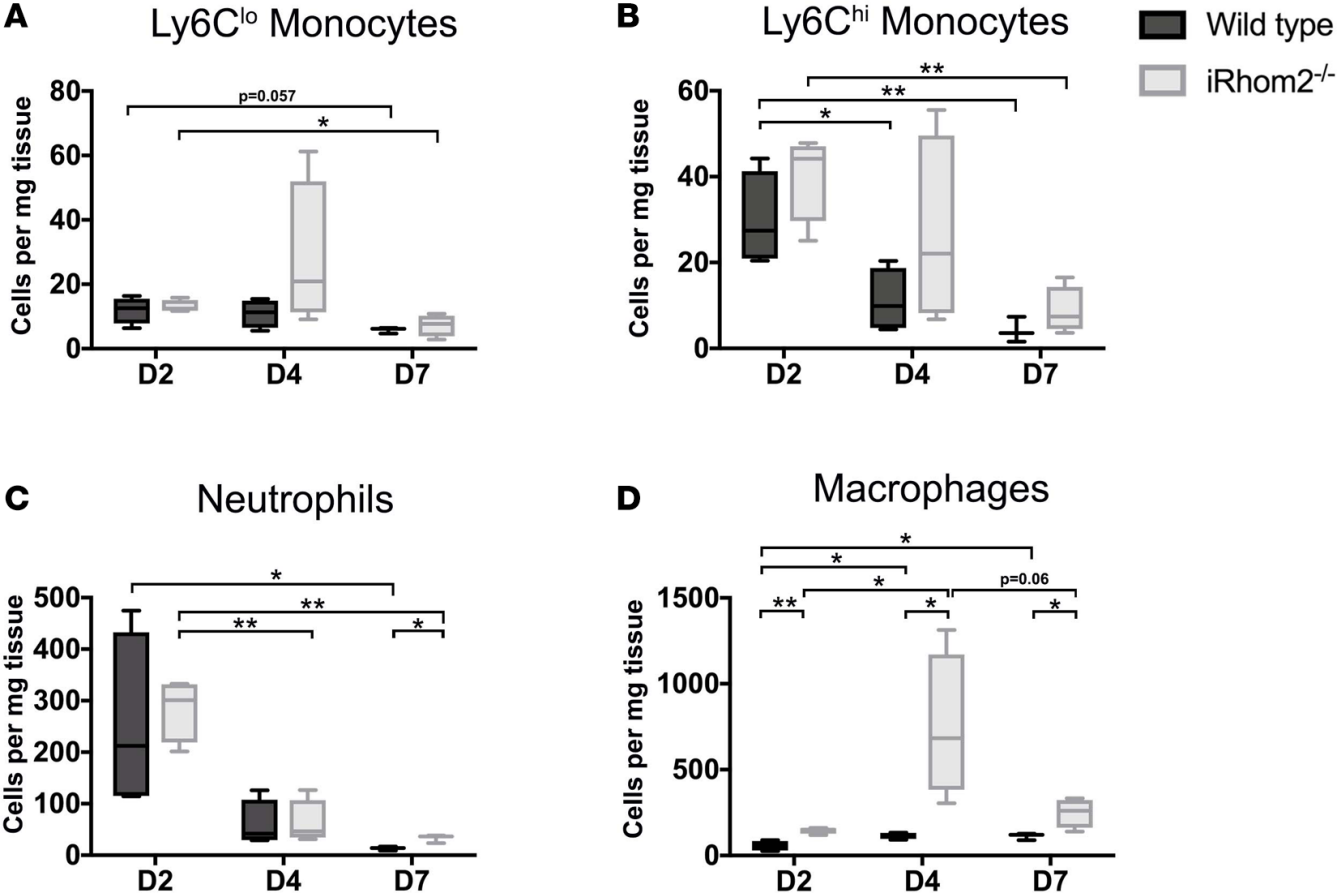
Immune responses in iRhom2-mutant adult mouse hearts after myocardial infarction. Bar graphs of immune cell populations distinguished by flow cytometry staining. (**A**) Reparative (CD45^+^CD64^+^MerTK^–^Ly6G^–^Ly6C^hi^) and (**B**) proinflammatory (CD45^+^CD64^+^MerTK^–^Ly6G^–^Ly6C^hi^) monocyte numbers were not significantly changed in iRhom2-deficient (iRhom2^–/–^) hearts compared with wild-type controls following injury. (**C**) Persistence of neutrophils (CD45^+^CD64^–^MerTK^–^Ly6G^+^) was observed in iRhom2 mutants, which is significant by 7 days after injury. (**D**) iRhom2^–/–^ mice exhibited an increase in the overall number of macrophages (CD45^+^CD64^+^MerTK^+^Ly6G^–^F4/80^+^) at day 2, 4, and 7 after myocardial infarction compared with wild-type mice. Data are shown as the mean ± SEM, *n* = 5 per experimental group. *P ≤ 0.05, **P ≤ 0.01, 1-way ANOVA and post-hoc test.

**Figure 2:**
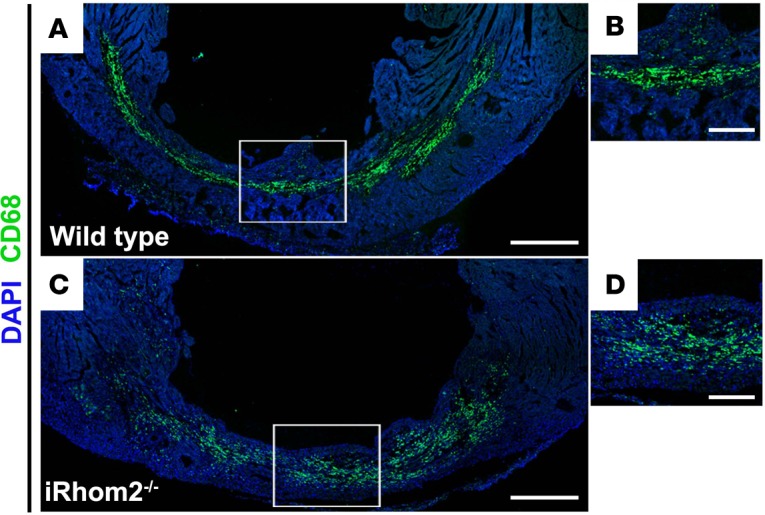
Localization of CD68^+^ immune cells within the infarct region of iRhom2-deficient (iRhom2^–/–^) hearts at day 7 after myocardial infarction (MI). Seven days after MI, (**A** and **B**) wild-type hearts revealed CD68^+^ myeloid cells (green) densely localized and highly organized within the infarct region. However, (**C** and **D**) iRhom2 mutants had an expression pattern of CD68^+^ immune cells that were more dispersed throughout the infarct zone. Representative images are based on 3 biological replicates. Scale bars: 500 μm (**A** and **C**), 200 μm (**B** and **D**).

We initially confirmed the loss of TNF-α secretion in bone marrow–derived MΦs (BMDMs) isolated from *Rhbdf2^–/–^* mice under basal conditions and proinflammatory stimulation with LPS/IFN-γ (Supplemental Figure 1A; supplemental material available online with this article; https://doi.org/10.1172/jci.insight.98268DS1). While TACE and its associated sheddase family members are coexpressed in a wide range of somatic tissues and have various cell surface targets (19), we did not observe any significant changes in the cell surface expression of other known substrates of iRhom2/TACE, including Tmem173 (also known as STING) and Csf1r, in resident cardiac MΦs (Supplemental Figure 1B) (20–22). iRhom2-deficient mice have previously been shown to elicit their effects specifically via myeloid cells due to the absence iRhom1 compensation (17, 18). Therefore, we verified reduced iRhom1 expression in cardiac MΦs compared with other cardiac cell types, which expressed similar levels of iRhom1 and iRhom2 (Supplemental Figure 1C), further confirming the myeloid specificity in the context of the heart. To analyze the effect of iRhom2 loss on immune cell recruitment after injury, we examined the inflammatory profiles of key immune cell populations (neutrophils, Mos, MΦs) involved in repair following heart injury by flow cytometry (Supplemental Figure 2). The resulting numbers of Ly6C^lo^ (CD45^+^CD64^+^MerTK^–^Ly6G^–^Ly6C^lo^) and Ly6C^hi^ (CD45^+^CD64^+^MerTK^–^Ly6G^–^Ly6C^hi^) Mos were not significantly changed in hearts from iRhom2-deficient animals compared with wild type during the first week of inflammation following injury (Figure 1, A and B). However, there was a significant persistence of neutrophils (CD45^+^CD64^–^MerTK^–^Ly6G^+^) in iRhom2-deficient mice versus controls at day 7 after injury (Figure 1C). Notably, the number of CD45^+^CD64^+^MerTK^+^Ly6G^–^F4/80^+^ MΦs increased in iRhom2-deficient animals compared with controls throughout the inflammatory phase, despite the apparently stable Mo numbers (Figure 1D). Interestingly, immune cells from iRhom2-deficient hearts lacked cell surface expression of the myeloid marker CD11b, although iRhom2-deficient and wild-type Mo/MΦ populations expressed comparable levels of classic markers including F4/80 (Supplemental Figure 3). Mononuclear cells from iRhom2-deficient mice also failed to organize properly within the infarct zone at day 7, as indicated by the dispersion of mutant CD68^+^ cells compared with a more concentrated localization within the core of the infarct in control mice (Figure 2, A and C). These changes in the inflammatory response of iRhom2-deficient mice following MI were attributed to alterations in their response to injury since uninjured iRhom2-deficient animals had no observable heart phenotype, and were morphologically equivalent to their wild-type littermates, with no changes in their immune cell profile (Supplemental Figure 4).

### iRhom2 is required for collagen deposition and efficient immune clearance during late-stage repair.

**Figure 3:**
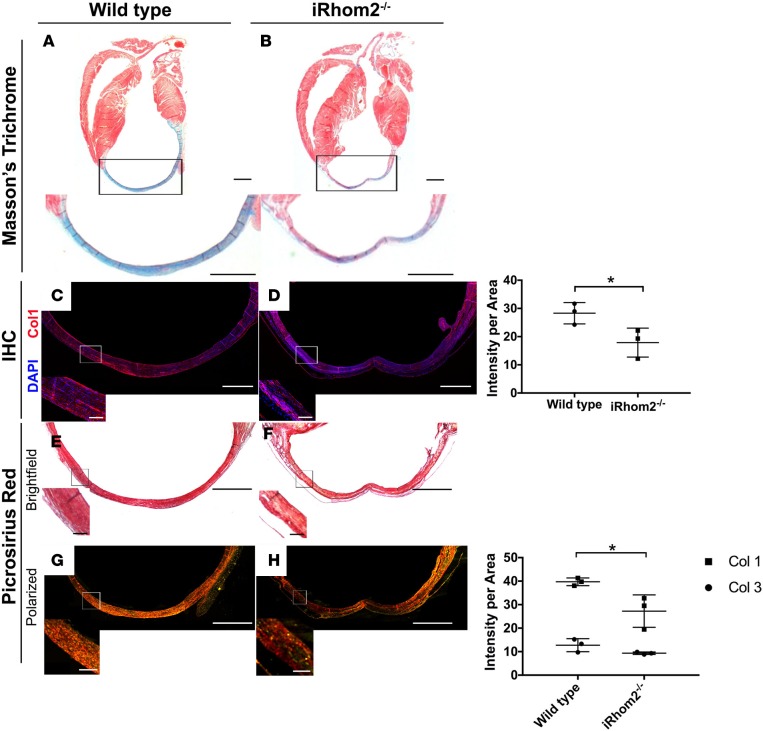
Collagen fiber deposition in the infarct region of iRhom2-deficient hearts at day 21 after myocardial infarction (MI). (**A** and **B**) Masson’s trichrome staining revealed wild-type hearts with strong collagen expression in the infarct region, with minimal viable myocardial tissue remaining 21 days after injury. However, iRhom2-deficient (iRhom2^–/–^) hearts had reduced collagen staining within the scar, with evidence of viable myocardium (arrows). Representative images are based on 3 biological replicates. (**C** and **D**) Immunohistochemistry showed a significant reduction in type I collagen expression in the scar of iRhom2^–/–^ hearts compared with wild-type hearts, and (right panel) collagen expression was quantified. (**E** and **F**) Picrosirius red staining imaged using bright-field microscopy showed a less uniform distribution of collagen in iRhom2^–/–^ hearts at 21 days after MI compared with controls. (**G** and **H**) Polarized-light microscopy revealed reduced mature type I collagen (red) in iRhom2^–/–^ compared with control injured hearts, while no differences in immature type III collagen (green) were observed, (right panel), which was quantified. Scale bars: 1 mm and 100 μm (insets). Data are shown as the mean ± SEM, *n* = 3 per experimental group. *P ≤ 0.05, Student’s *t* test.

**Figure 4:**
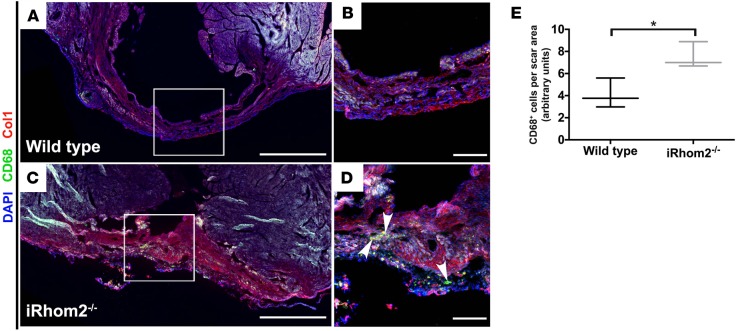
Presence of immune cells within the scar of iRhom2-deficient hearts at 28 days after myocardial infarction (MI). Immunofluorescence of infarcted hearts 28 days after MI showed (**A** and **B**) reduced immune cells in wild-type hearts, while (**C** and **D**) iRhom2-deficient (iRhom2^–/–^) hearts had persistent CD68^+^ immune cells (green, indicated by arrowheads) and disorganized type I collagen fibers (red) throughout the scar region after long-term repair. (**E**) Number of CD68^+^ cells within the infarct region was quantified relative to the scar area using ImageJ software. Scale bars: 1 mm (**A** and **C**) and 200 μm (**B** and **D**). Data are shown as the mean ± SEM, *n* = 3 per experimental group. *P ≤ 0.05, Student’s *t* test.

Given that iRhom2-deficient animals displayed an altered MΦ response during the early phases following cardiac injury, we sought to investigate the effect of these changes on downstream processes of repair such as scar formation and maturation. Hearts from iRhom2-deficient animals exhibited an overall reduction in collagen within the scar, compared with wild-type animals at 21 days after injury, as determined by Masson’s trichrome staining (Figure 3, A and B). Specifically, the levels of the most abundant collagen isoform in mature scars, collagen type I α 1 (Col1α1) (23), were significantly reduced within the infarct region of iRhom2-deficient hearts compared with controls (Figure 3, C and D). To further investigate scar composition, we used picrosirius red staining to examine collagen deposition and fiber alignment within the infarct region. iRhom2-mutant mice displayed an overall decrease in collagen deposition, with a less uniform distribution, appearing more punctate throughout a thin, fibrous scar (Figure 3, E and F). Subsequent application of polarized light microscopy enabled examination of both mature (type 1 collagen; Col1) and immature (type 3 collagen; Col3) isoforms, and confirmed that the scar composition in iRhom2-deficient injured hearts had reduced Col1 within the scar compared with wild-type littermates (Figure 3, G and H). This reduction in mature collagen fibers indicates a scar that lacks appropriate stiffness and rigidity and which consequently is prone to rupture. In addition, we examined the scar 1 week later, at day 28 after MI, and determined that iRhom2-deficient mice retained a significant number of CD68^+^ mononuclear cells within a disorganized matrix (Figure 4), suggestive of persistent inflammation and continued remodeling of the collagen and fibrous support during late-stage repair.

### iRhom2-deficient animals exhibit increased left ventricular remodeling and increased mortality following injury.

**Figure 5:**
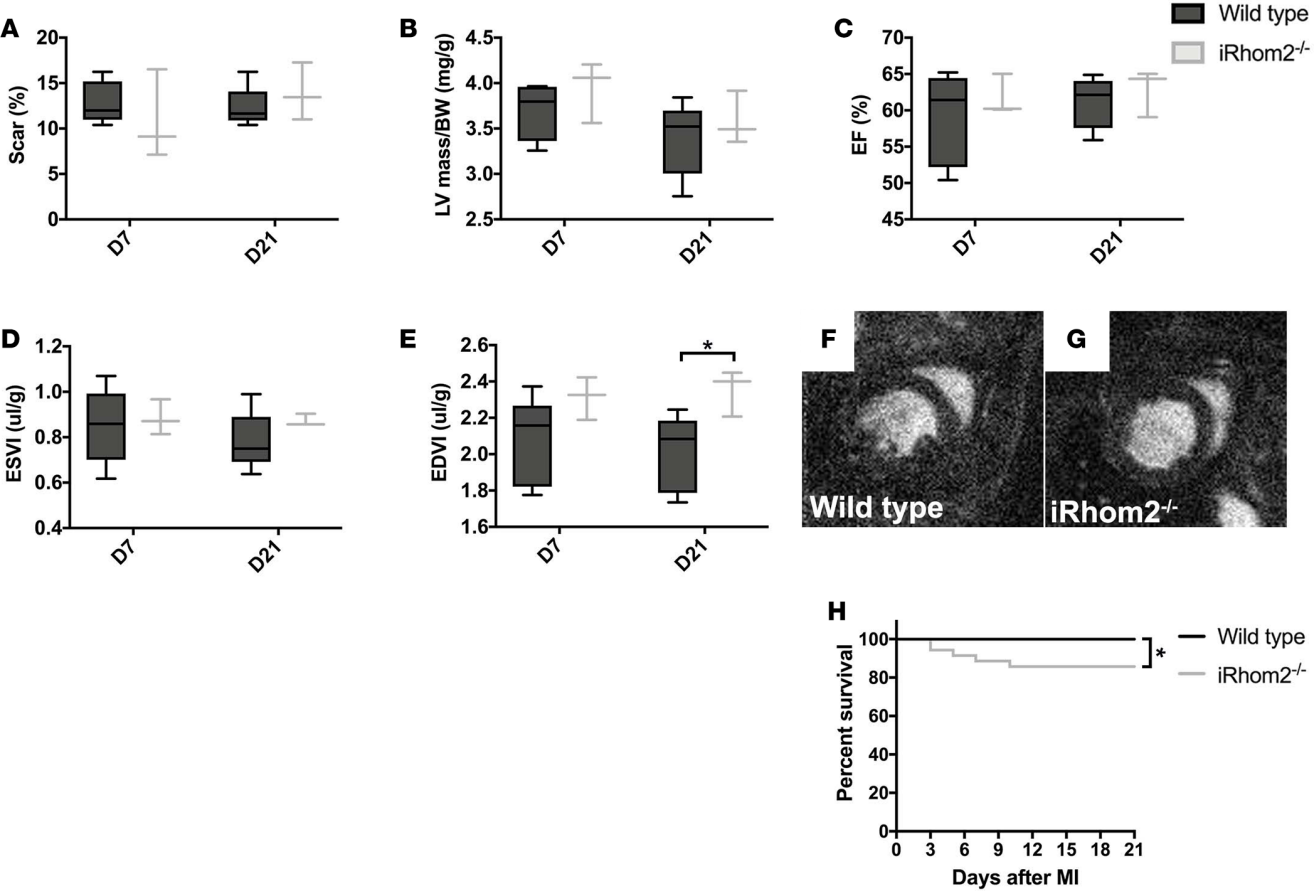
MRI functional parameters and Kaplan-Meier survival curve following myocardial infarction (MI). (**A**) Scar size measurements as a percentage of the left ventricle at day 7 (WT, 12.87 ± 2.32; iRhom2-deficient [iRhom2^–/–^], 10.92 ± 4.95) and day 21 (WT, 12.32 ± 2.26; iRhom2^–/–^, 13.92 ± 3.16) after MI. (**B**) Left ventricular mass normalized to body weight (LV/BW, mg/g) shows no changes between wild-type and iRhom2^–/–^ at day 7 (WT, 3.69 ± 0.31; iRhom2^–/–^, 3.94 ± 0.34) or day 21 (WT, 3.39 ± 0.41; iRhom2^–/–^, 3.58 ± 0.29). (**C**) Ejection fraction (EF, %) at day 7 (WT, 58.91 ± 6.43; iRhom2^–/–^, 61.78 ± 2.81) and day 21 (WT, 61.07 ± 3.55; iRhom2^–/–^, 62.79 ± 3.26) are comparable. (**D**) Left ventricular end-systolic volume (ESV) normalized to body mass (ESVI, μl/mg) at day 7 (WT, 0.85 ± 0.17; iRhom2^–/–^, 0.88 ± 0.08) and day 21 (WT, 0.78 ± 0.13; iRhom2^–/–^, 0.87 ± 0.03) after MI. (**E**) Normalized left ventricular end-diastolic volume (EDVI, μl/mg) show no changes at day 7 (WT, 2.07 ± 0.24; iRhom2^–/–^, 2.31 ± 0.12), but a significant increase in iRhom2^–/–^ animals by day 21 (WT, 2.01 ± 0.21; iRhom2^–/–^, 2.35 ± 0.13). (**F** and **G**) Representative longitudinal short-axis MRI analyses in diastole of infarcted hearts 21 days following MI from (**F**) wild-type and (**G**) iRhom2^–/–^ animals. (**H**) Kaplan-Meier survival curves during the first 21 days after MI show decreased survivability of iRhom2^–/–^ animals compared with wild-type controls (*n* ≥ 25). Data are shown as the mean ± SEM, *n* = 5 for MRI analyses, and *n* ≥ 25 for Kaplan-Meier survivability. *P ≤ 0.05, 1-way ANOVA and post-hoc test.

To further explore the influence of an altered immune cell milieu and improper scar formation on cardiac function, we examined wild-type and iRhom2-deficient animals at day 7 and day 21 after injury using longitudinal MRI. We observed that iRhom2-deficient mice had a scar size and left ventricular (LV) mass index comparable to wild-type animals (Figure 5, A and B), which suggests that loss of iRhom2 does not affect the area at risk or acute ventricular response to injury. Likewise, the contractility and overall function of viable myocardium was similar between wild-type and iRhom2-deficient mice, as indicated by no changes in end-systolic volume (ESV) and ejection fraction (EF; Figure 5, C and D). However, during diastole we observed a significant increase in the end-diastolic volume (EDV) in iRhom2-deficient hearts compared with controls at day 21 after MI (WT, 2.01 ± 0.21 μl/g; iRhom2-deficient, 2.35 ±0.13 μl/g; *n* = 5 mice per group; mean ± SEM; *P* ≤ 0.05; Figure 5E), which was visualized by an expanded lumen adjacent to the scar of the ventricular wall (Figure 5, F and G). This suggests that iRhom2-deficient mutants undergo elevated pathological remodeling, consistent with impaired scar maturation and reduced wall resistance during the cardiac cycle when blood pressure and volumes are high, and supports the impaired fibrillar collagen deposition and scar density observed by histology (Figure 3, A–H). The increased EDV and LV wall thinning was accompanied by a significant increase in mortality in iRhom2-deficient mice after MI (20% loss by day 21) as illustrated by a Kaplan-Meier survival curve (Figure 5H).

### MΦs deficient in iRhom2 signaling lose their proinflammatory and reparative phenotypes during repair.

**Figure 6:**
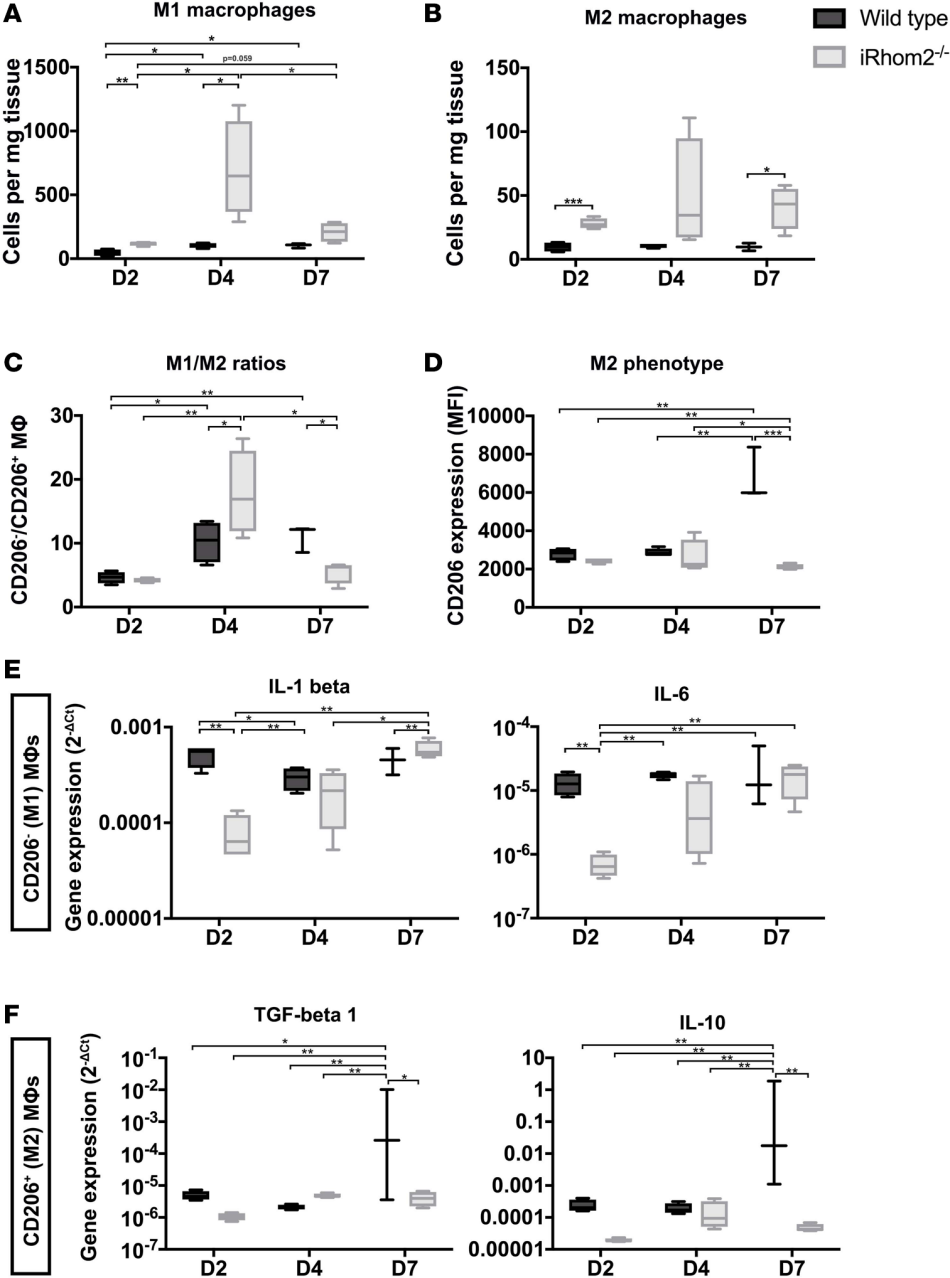
M1/M2 macrophage ratios and cytokine expression within the first week after injury. The numbers of (**A**) CD206^–^ M1 macrophages (MΦs) were significantly increased at days 2 and 4 in iRhom2-deficient (iRhom2^–/–^) hearts compared with controls. Similarly, the numbers of (**B**) CD206^+^ M2 MΦs were increased significantly at days 2 and day 7 in iRhom2^–/–^ hearts. (**C**) M1/M2 ratios were increased and decreased at days 4 and 7, respectively, in iRhom2^–/–^ mutants compared with wild type. (**D**) The mean fluorescence intensity (MFI) of CD206^+^ M2 MΦs was markedly reduced in iRhom2^–/–^ MΦs at day 7 after injury. (**E**) Relative mRNA expression (2^–ΔCT^) of proinflammatory cytokines (*Il1b* and *Il6*) from sorted M1 MΦs (CD45^+^CD64^+^MerTK^+^Ly6G^–^F4/80^+^CD206^–^) was significantly decreased at day 2 after injury compared with wild-type controls. (**F**) Gene expression of reparative cytokines (*Il10* and *Tgfb1*) were significantly reduced in sorted M2 MΦs (CD45^+^CD64^+^MerTK^+^Ly6G^–^F4/80^+^CD206^+^) at day 7 after injury, compared with wild-type hearts. Data are shown as the mean ± SEM, *n* = 5 for each experimental group. *P ≤ 0.05, **P ≤ 0.01, ***P ≤ 0.001, 1-way ANOVA and post-hoc test.

To explain how early immune cell changes impact late-stage scar formation and compliance and functional outcome, we examined whether loss of iRhom2 alters the balance of classically defined proinflammatory (M1) versus antiinflammatory/reparative (M2) MΦs during early repair. Consistent with overall MΦ numbers, M1 (defined by CD206 negativity) and M2 (defined by CD206 positivity) MΦ subpopulations increased in iRhom2-deficient mice throughout the first week of repair following MI (Figure 6, A and B). Interestingly, we observed a significant increase in the M1/M2 MΦ ratio in mutant hearts at day 4 compared with wild-type controls, suggesting a skewed response towards proinflammatory MΦs; followed by a decrease in the M1/M2 ratio at day 7, indicative of overall more reparative MΦs (Figure 6C). However, coincident with this altered cell ratio profile we observed a strong reduction in the overall cell surface expression of the mannose receptor CD206 in MΦs from iRhom2-deficient mice at day 7 relative to wild-type controls (Figure 6D). Since the widely established classification of M1/M2 MΦ populations is largely based on nonfunctional markers, we assessed the expression of functional cytokines in these 2 MΦ subpopulations to establish a clear readout of MΦ phenotypes. We determined that iRhom2-deficient cardiac MΦs have decreased expression of proinflammatory cytokines (*Il1b* and *Il6*) during the initial inflammatory response (day 2) compared with controls (Figure 6E), signifying impaired M1 function. In addition, we also observed a decrease in the downstream reparative cytokines (*Tgfb* and *Il10*) in iRhom2-deficient MΦs at later stages of inflammation (day 7) (Figure 6F). These data suggest that early, proinflammatory and late, reparative MΦ phenotypes are iRhom2 dependent, which in turn impacts on subsequent scar formation and wound healing. These findings show iRhom2 signaling substantially influences both M1 and M2 MΦ phenotypes through an effect on the proinflammatory cascade, in contrast to previous findings suggesting antiinflammatory signaling via M2 MΦs alone determines infarct repair (24).

### Loss of iRhom2 alters BMDM phenotypes and diminishes their function.

**Figure 7:**
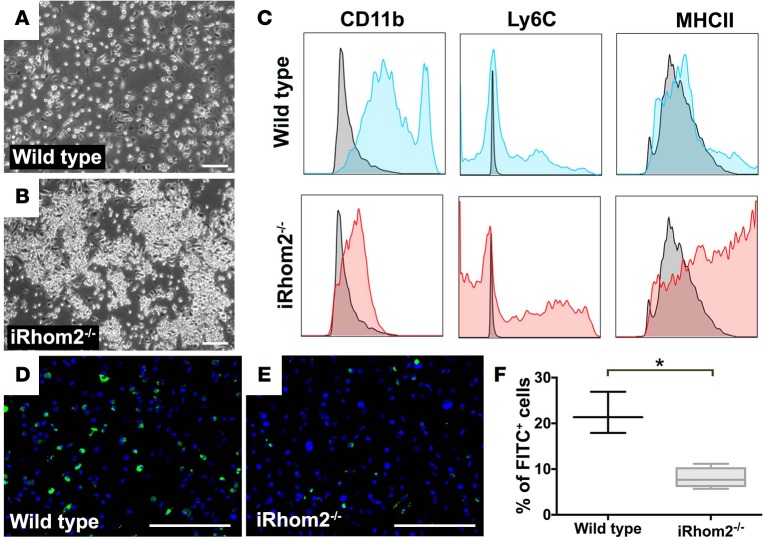
Immunophenotyping of BMDMs from iRhom2-deficient mice. Bone marrow cells were differentiated in conditioned media for 7 days to generate bone marrow–derived macrophages (BMDMs). Bright-field images revealed (**A**) wild-type cultures form a dispersed monolayer of cells, (**B**) while iRhom2-deficient (iRhom2^–/–^) BMDM cultures formed clusters of cell aggregates (arrowheads). (**C**) Flow cytometry shows F4/80^+^ macrophages expressed moderate to high levels of the myeloid differentiation maker CD11b (MFI: 42,758 ± 2,955). However, F4/80^+^ iRhom2^–/–^ cells expressed low levels of CD11b (MFI: 1,266 ± 64) and increased expression of the monocyte marker Ly6C (MFI: 10,631 ± 716), as well as an increased expression in the late-stage monocyte marker class II MHC (MFI: 34,324 ± 3,687), as compared with wild-type BMDMs (Ly6C MFI: 3,198 ± 1,150; MHCII MFI: 12,733 ± 3,814). (**D**–**F**) Phagocytosis assays revealed iRhom2-deficient BMDMs were functionally impaired and failed to phagocytose FITC-labeled latex beads as efficiently as control BMDMs. Scale bars: 200 μm. *n* = 3 for each experimental group. Data are shown as the mean ± SEM, *P ≤ 0.05, Student’s *t* test. MFI, mean fluorescence intensity.

A large proportion of MΦs in the infarct zone after cardiac injury originate from infiltrating bone marrow–derived blood Mos that differentiate into MΦs once in situ (25). To study the effect of iRhom2 function in an isolated system, devoid of other exogenous signaling factors, we examined the phenotypic response to stimulation of iRhom2-deficient BMDM cultures in vitro. After maintaining bone marrow cells for 7 days under differentiation conditions, BMDMs from iRhom2-deficient mice appeared morphologically immature (26), and formed large aggregates compared with wild-type cells that formed a dispersed monolayer (Figure 7, A and B). This suggests that iRhom2-deficient bone marrow cells may have a defect in MΦ differentiation, consistent with the reduced cytokine profiles we observed in the cardiac population. Therefore, we examined MΦ precursor markers by flow cytometry and found that iRhom2-deficient BMDMs (CD45^hi^F4/80^hi^), similar to cardiac MΦs, did not express CD11b (mean fluorescence intensity [MFI]: 1,266 ± 64), while the majority of wild-type BMDMs demonstrated either intermediate (CD11b^int^) or high (CD11b^hi^) expression (MFI: 42,758 ± 2,955) (Figure 7C). In addition, a significant number of iRhom2-deficient BMDMs expressed higher levels of the MΦ precursor markers Ly6C (MFI: 10,631 ± 716) and MHC class II (MFI: 34,324 ± 3,687), compared with wild-type expression (Ly6C MFI: 3,198 ± 1,150; MHCII MFI: 12,733 ± 3,814). Given the dampened phenotypes of iRhom2-deficient MΦs observed in vivo and in vitro, we next tested their phagocytic function. We determined that 22% ± 3% (mean ± SEM; *n* = 3 cultures) of wild-type BMDMs were able to phagocytose FITC-labeled latex beads (Figure 7D), compared with only 7% ± 2% (mean ± SEM; *n* = 3 cultures) of iRhom2-deficient BMDMs (Figure 7E), which was a significant reduction in phagocytosis when quantified across independent experiments (Figure 7F). These observations suggest that blood-derived MΦs that accumulate in the heart after injury require iRhom2 to terminally differentiate and phagocytose dying cells, including neutrophils, which likely explains the abnormal persistence of the latter in iRhom2-mutant hearts at day 7 after MI.

### iRhom2-deficient BMDMs can respond to polarization stimuli and are sensitized to exogenous TNF-α treatment.

**Figure 8:**
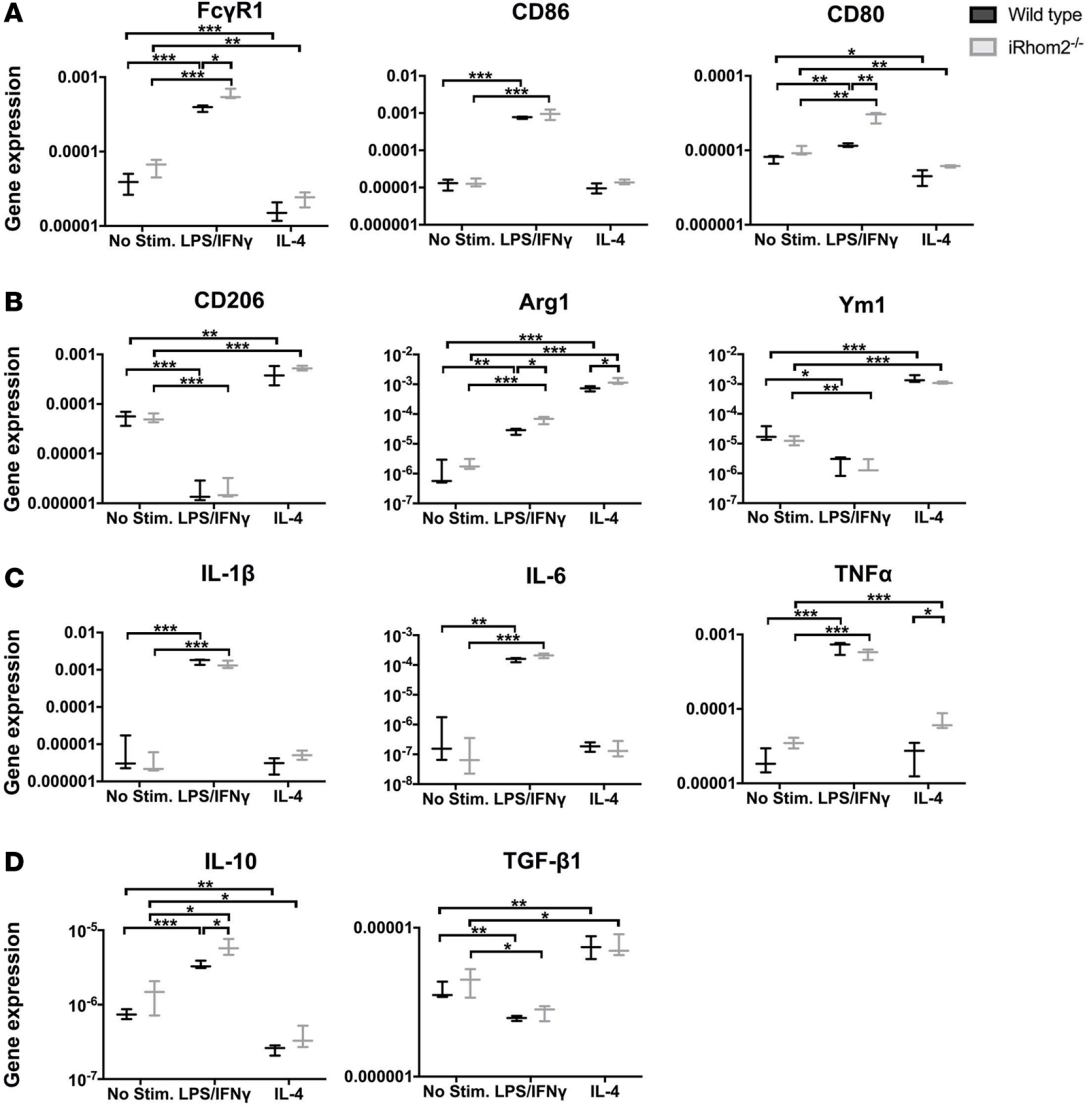
Polarization and cytokine activation of BMDMs after in vitro stimulation. Bone marrow–derived macrophages (BMDMs) were stimulated for 16 hours with either LPS/IFN-γ or IL-4 to promote M1- or M2-like macrophages (MΦs), respectively. (**A**) iRhom2-mutant cells had increased M1 gene expression (*Fcgr1* and *Cd86*) when stimulated with LPS/IFN-γ compared with controls, and reduced inhibition (*Cd80*) with IL-4 antiinflammatory stimulation. (**B**) Stimulation of iRhom2-deficient (iRhom2^–/–^) BMDMs with IL-4 activated M2 gene expression (*Cd206* and *Arg1*) to a significantly greater extent than in wild-type controls. (**C**) iRhom2-mutant cells had increased M1 cytokines when treated with proinflammatory stimulation LPS/IFN-γ similar to controls, but relatively increased TNF-α expression with IL-4 stimulation. (**D**) iRhom2^–/–^ MΦs revealed similar induction of TGF-β1, and increased IL-10, under proinflammatory (LPS/IFN-γ) conditions, compared with control cells. Data are shown as the mean ± SEM, *n* = 3 for each experimental group. *P ≤ 0.05, **P ≤ 0.01, ***P ≤ 0.001, 1-way ANOVA and post-hoc test.

**Figure 9:**
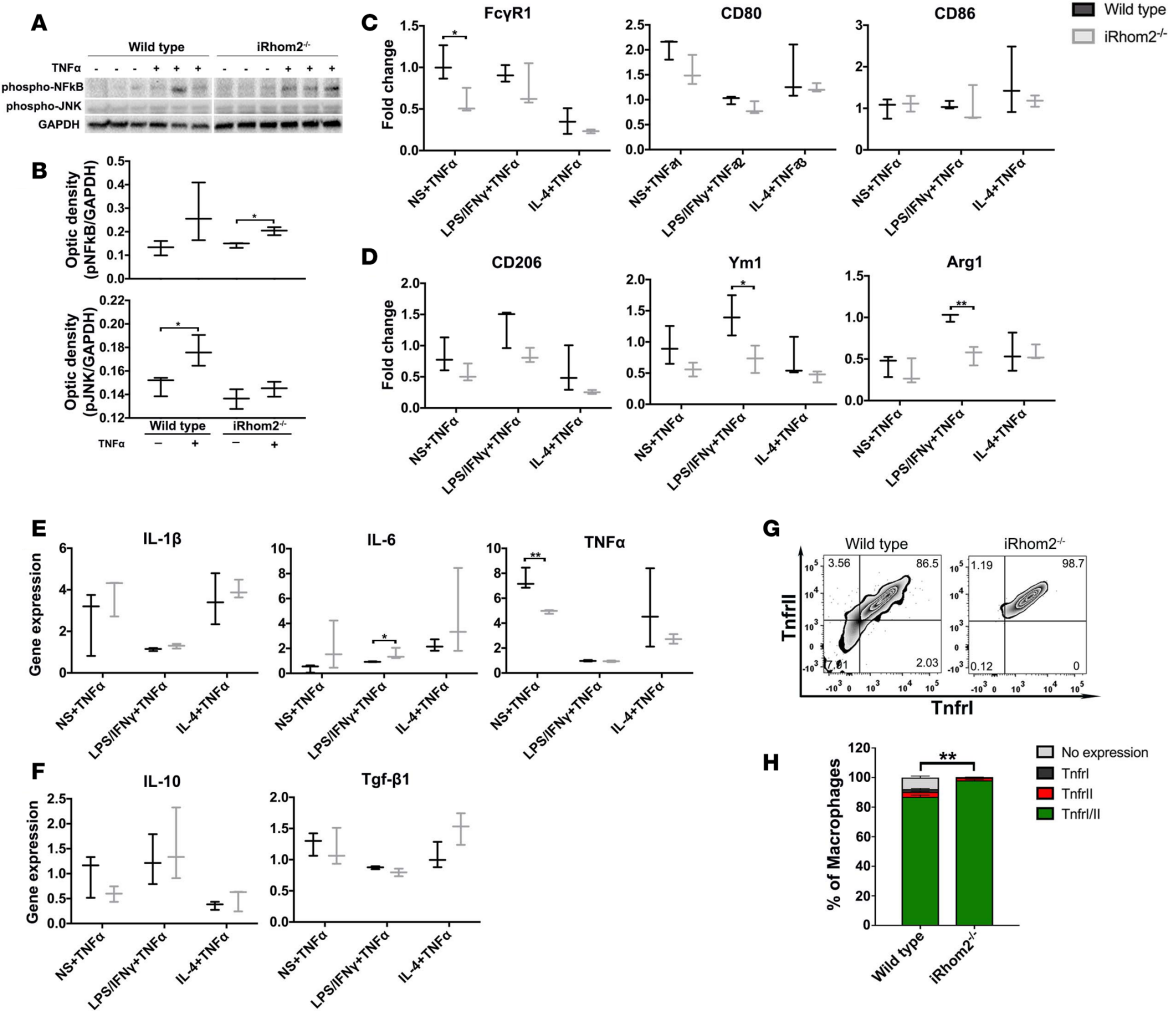
BMDM responsiveness, polarization, and cytokine expression after exogenous TNF-α stimulation. (**A**) Western blotting showed that iRhom2-deficient (iRhom2^–/–^) bone marrow–derived macrophages (BMDMs) treated with 20 ng/ml TNF-α display activation of TNF-α downstream signaling protein phospho-NF-κB (p-NF-κB), while wild-type BMDMs displayed increased levels of p-NF-κB and p-JNK. Wild-type and iRhom2^–/–^ samples were run in the same gel but were noncontiguous. (**B**) Scanning densitometry shows significant increases in p-NFκB and p-JNK relative to the loading control GAPDH. Data are shown as the mean ± SEM, *n* = 3 for each experimental group.*P ≤ 0.05, Student’s *t* test. TNF-α–stimulated iRhom2^–/–^ BMDMs show significantly reduced (**C**) M1 and (**D**) M2 marker expression compared with controls, while (**E**) M1 and (**F**) M2 cytokine expression was partially affected. Gene expression normalized to respective stimulations without TNF-α treatment. Flow cytometric analysis of iRhom2^–/–^ BMDMs: (**G**) A contour plot of the number of macrophages expressing TNFRI (MFI: 5,737 ± 59) and/or TNFRII (MFI: 14,615 ± 112) compared with wild-type BMDMs (TNFRI MFI: 3,830 ± 340; TNFRII MFI: 8,456 ± 251), and (**H**) biological replicates were quantified. Data are shown as the mean ± SEM, *n* = 4 for each experimental group. **P ≤ 0.01, 1-way ANOVA and post-hoc test. MFI, mean fluorescence intensity; TNFRI, type I TNF receptor; TNFRII, type II TNF receptor.

We next examined the ability of iRhom2-deficient MΦs to respond to proinflammatory (LPS/IFN-γ) and antiinflammatory (IL-4) stimuli. Surprisingly, iRhom2-deficient BMDMs were able to induce expression of classic M1 markers with LPS/IFN-γ stimulation to a greater extent than wild-type BMDMs, as observed by significant relative increases in *Fcgr1* and *Cd80* gene expression (Figure 8A). Similarly, IL-4 stimulation resulted in relatively increased induction of M2 markers in iRhom2-deficient BMDMs compared with controls, trending towards significance (Figure 8B). To assess definite MΦ function, we examined cytokine gene profiles and observed a similar increase in proinflammatory cytokines (*Il1b*, *Il6*, *Tnfa*) in both wild-type and iRhom2-deficient BMDMs when stimulated with LPS/IFN-γ (Figure 8C). Reparative cytokines were induced in iRhom2-deficient MΦs, with IL-10 being increased above controls (Figure 8D). Collectively, these data suggest that iRhom2-deficient MΦs are responsive to proinflammatory and antiinflammatory stimuli, to an extent comparable to that of their wild-type counterparts, in an in vitro system lacking soluble signaling factors. Since TNF-α is an established key determinant of (pro-)inflammation following cardiac injury and a major target of the iRhom2/TACE pathway, we examined whether iRhom2-deficient BMDMs are competent to respond to exogenous TNF-α. While iRhom2-deficient MΦs cannot produce autocrine TNF-α, the latter is produced by a variety of cell types including cardiomyocytes and fibroblasts in the injured heart, which in turn can signal to the infiltrating MΦ population (27–29). We determined that iRhom2-deficient MΦs are able to significantly activate the downstream NF-κB pathway following TNF-α treatment, although we did not observe an equivalent activation of the JNK pathway, as was evident in activated wild-type BMDMs (Figure 9, A and B). In addition, exogenous TNF-α inhibited the ability of iRhom2-deficient BMDMs to activate M1 and M2 genes, with or without stimulation, compared with wild-type BMDMs when normalized to respective stimulations in the absence of TNF-α (Figure 9, C and D). This was also associated with subtle changes in signature cytokines (Figure 9, E and F). These data suggest that iRhom2-deficient BMDMs are sensitive to exogenous TNF-α, but downstream signaling is partially perturbed at the level of the JNK pathway and M1/M2 gene expression. To explain the apparent response to exogenous TNF-α via elevated NK-κB signaling, we observed a significant increase in the cell surface expression of membrane-bound TNFRI (MFI: 5,737 ± 59) and TNFRII (MFI: 14,615 ± 112) on iRhom2-deficient BMDMs compared with those from wild-type BMDMs (TNFRI MFI: 3,830 ± 340; TNFRII MFI: 8,456 ± 251) (Figure 9, G and H). TNFRs are themselves targets of TACE for recycling, and in the absence of iRhom2 remain membrane bound and able to differentially regulate downstream targets in response to external ligand via autocrine signaling (30). Addition of exogenous TNF-α to iRhom2-deficient BMDMs resulted in increased aggregation of cells and elevated numbers of Ly6C^hi^/MHCII^hi^ Mo derivatives (Supplemental Figure 5), consistent with enhanced TNF-α signaling due to accumulation of cell surface TNFRs. Together, these data demonstrate that iRhom2 regulates TNF-α responsiveness, polarization, and cytokine profiles in MΦs, all of which are essential parameters for optimal heart repair and function after injury.

## Discussion

The role of TNF-α in orchestrating proinflammatory responses in tissue injury and disease has been well described. However, little is known about the immune cell contribution of TNF-α in this context. Studies investigating the proinflammatory response after MI have focused on TNF-α signaling and its effect on late-stage remodeling, particularly infarct size and cardiac output (10, 12, 13), but have not defined a role for TNF-α in influencing cardiac immune cell phenotypes or function. Here, we establish for the first time to our knowledge that iRhom2 regulates TNF-α signaling and proinflammatory cues in classically activated M1 MΦs, which acts as a significant determinant of the downstream repair process to mediate collagen composition and alignment in the scar following MI (Figure 3). The iRhom2-deficient mouse model offered insight into the requirements of MΦ TNF-α signaling at 2 levels: first, the ability to release soluble TNF-α within the surrounding tissue to promote proinflammation, and second, the ability to respond to TNF-α produced by neighboring cardiovascular cells. iRhom2-mutant macrophages were unable to cleave both membrane-bound TNF-α and its associated receptors (TNFRI and TNFRII); thus, they were unable to downregulate the effects of paracrine TNF-α signaling to promote antiinflammation and effective repair. The net effect of impaired TNF-α signaling in injured iRhom2-deficient hearts was increased MΦ numbers during the inflammatory response, and persistent neutrophils until day 7 after injury (Figure 1). Notably, Mo numbers in iRhom2-deficient hearts were unaltered during the early phase of inflammation (up to day 2 after MI), suggesting that iRhom2-mediated TNF-α signaling from myeloid cells is not required for immune cell recruitment and/or retention.

The function of MΦs in repair following MI has been studied extensively (31, 32). Several reports have shown that Mo/MΦ infiltration is required for post-MI healing, which is facilitated by their ability to phagocytose dying cells and secrete trophic and profibrotic cytokines (2). Moreover, it is now well established that MΦ polarization into either proinflammatory M1 or antiinflammatory/reparative M2 subpopulations regulates their function (32). In post-MI iRhom2-deficient MΦs, the overall ratio of M1/M2 MΦs residing in injured iRhom2-deficient hearts, as classically defined by the absence or presence of CD206 expression, respectively, was increased at day 4 and decreased at day 7 (Figure 6C), indicating more M1 and M2 macrophages, respectively. However, the more functionally relevant M1 proinflammatory cytokine signature was significantly dampened (*Il1b* and *Il6*) at the initiation of inflammation, associated with a subsequent reduction in CD206 expression and M2 reparative cytokine expression (*Il10* and *Tgfb*) during later stages (Figure 6, E and F). These data suggest that MΦ polarization towards M1 during the early immune response is mediated by the iRhom2/TNF-α proinflammatory signaling axis (33, 34), and this targeted effect impacts on the incidence, polarization, and function of later-stage M2 MΦs (Figure 6). There are conflicting findings on the significance of a shift toward M2-like MΦs with regards to scar formation, matrix remodeling, and ventricular function after MI (24, 35–37). A study examining TRIB1 kinase–deficient mice, which are depleted for M2 MΦs, revealed that reparative signaling and antiinflammatory cytokine IL-4 appear to be critical for activating cardiac fibroblasts and the ensuing fibrosis for effective repair of the infarcted heart (24). Alternatively, here we reveal that scar formation and wound healing are dependent on proinflammatory signaling pathways, particularly TNF-α, which are characteristic of the M1 MΦ population. The differences between findings may reflect the oversimplification of the M1 and M2 classification as 2 distinct populations. M1 and M2 signatures are not necessarily mutually exclusive, suggesting that there is no formation of stable subsets. Instead, activated MΦs mount a graded response to a combination of factors present in injured tissues and adopt complex pro- versus antiinflammatory phenotypes, which are often mixed (38).

The infarct scars in iRhom2-deficient hearts at late-stage remodeling had a reduction in overall collagen (Figure 3), and the persistence of immune cells within the scar (Figure 4). This suggests a more inflamed and unstable scar, which is prone to cardiac rupture. Consistent with this, our functional studies revealed that iRhom2-deficient mice had increased LV remodeling, indicative of impaired wound healing, with wall thinning and dilatation proximal to the scar region that ultimately resulted in an increased mortality (Figure 5). Interestingly injured iRhom2-deficient hearts revealed a preserved cardiac output after MI, consistent with them being on a 129S6 background, which is known to have higher EF at baseline and following injury (39). Inhibition of TNF-α signaling has previously resulted in adverse remodeling associated with increased incidence of rupture (13); however, the underlying cellular mechanism was not reported. A recent study suggested that TNF-α exerts disparate effects through distinct receptor signaling (40). TNFRI signaling has been proposed to be detrimental to outcome after MI, whereby inhibition of the pathway was associated with improved survival; while TNFRII signaling is thought to be protective by mitigating adverse remodeling. Moreover, TNFRI/II signaling can be activated both by soluble and membrane-bound TNF-α through interactions with neighboring cells (41). We observed higher levels of cell surface TNFRI/II in iRhom2-deficient MΦs leading to enhanced activation of proinflammatory cytokines in response to TNF-α (Figure 9). This suggests that the iRhom2-deficient MΦs are sensitized to ectopic TNF-α through enhanced, differential TNFR signaling. In macrophages, TNF-α has been shown to activate JNK via TNFRI signaling to induce production of TGF-β1 (42), and the latter plays a key role in myofibroblast activation and subsequent collagen expression and fibrosis (23, 43, 44); however, TNFRII signaling has been shown to inhibit TNFRI-induced JNK activation (45, 46). In this iRhom2 model, the effect on JNK activation is specific to the TNF-α downstream signaling, given that other TACE-substrate pathways (such as Csf1r and Tmem173/STING) are not known to phosphorylate JNK. This TNFR crosstalk establishes a causal link between increased surface TNFRII on cardiac MΦs (Supplemental Figure 1), reduced JNK activation (Figure 9), and impaired fibrosis (Figure 3) observed in iRhom2-deficient animals following MI.

The innate immune response to cardiac injury is a prerequisite for effective wound healing and 2 discrete phases have been defined as proinflammatory and antiinflammatory/reparative according to the spatiotemporal infiltration and in situ polarization of Mos and MΦs. Our findings of a potentially novel requirement for iRhom2 and myeloid cell–derived TNF-α signaling in optimizing fibrosis and scar formation suggests that the proinflammatory MΦ phenotype is an important determinant of infarct repair, and that the later reparative phase is not exclusively regulated by the antiinflammatory cues attributed to M2 macrophages, as previously suggested (24). The implication of these findings is one of crosstalk between the proinflammation and repair stages following MI, which prompts a reevaluation of the existing biphasic model (6, 47). Consequently, there is a need to appreciate the heterogeneity and range of phenotypic and functional plasticity of MΦs during the injury response, and to redefine the M1/M2 classification (38, 48).

Attributing a role for TNF-α in fibrosis and repair has implications for chronic inflammation and disease, and may explain the relative failure of clinical trials of anti–TNF-α therapy with respect to cardiovascular indications. In patients with rheumatoid arthritis, who are at risk of cardiovascular disease, anti–TNF-α treatment has been shown to be protective but also, in some cases, to increase the risk of MI (49). Moreover, large multicenter trials of anti–TNF-α therapy in heart failure patients have not only failed to demonstrate clinical benefit, but suggest targeting TNF-α blockade may adversely affect the course of the disease (50). The mechanistic basis for the failure of these trials, and why targeting TNF-α may be detrimental, is unknown. However, effective repair will be critical to counter the risk of MI events or progression from moderate to severe heart failure in the patient populations. Our findings suggest that alternative strategies should be evaluated to alleviate the persistent inflammation associated with chronic cardiovascular disease.

## Methods

### Animals.

Eight- to ten-week-old mice were used in the experiments. *Rhbdf2^–/–^* mice (SV/129 background) were provided by Matthew Freeman (University of Oxford, UK). SV/129 mice were purchased from Charles River Laboratories, and used as age-matched, wild-type controls.

### Myocardial infarction surgery.

*Rhbdf2^–/–^* and control mice were subject to ligation of the left anterior descending (LAD) artery, as previously described (51). In brief, mice were anesthetized, intubated for assisted ventilation, and permanent ligation was achieved by tying a suture around the LAD. Hearts were collected at 2, 4, 7, 21, and 28 days after injury, and harvested tissue was analyzed by flow cytometry, histology, or immunofluorescence. All surgical procedures were performed in accordance with the Animals (Scientific Procedures) Act 1986 (Home Office, UK).

### Single-cell isolation of cardiac cells.

Murine heart cells were isolated as previously described (52). Immediately after cervical dislocation, hearts were excised and placed in cold HBSS (Sigma-Aldrich). Isolated hearts were finely minced into small pieces, and digested with collagenase type II (Worthington Laboratories) solution (containing 500 units/ml HBSS) at 37°C for 30 minutes with agitation. Supernatant was removed and 10% heat-inactivated FBS (Sigma-Aldrich) was added, and the remaining tissues were digested with a fresh collagenase solution for a total of 3 times. Cell suspensions were combined and filtered through a 40-μm cell strainer (BD Falcon). Cells were centrifuged, washed with PBS, and Red Cell Lysis buffer (BioLegend) used according to the manufacturer’s instructions to remove red blood cells. The single cardiac cells were stained and subjected to flow cytometric analyses or FACS.

### Immunohistochemistry.

Hearts were collected at 7, 21, and 28 days after MI, and fixed in 4% paraformaldehyde solution overnight at 4°C. Heart tissue was processed for paraffin embedding, sectioned, and deparaffinized. Sections were blocked in serum from the same species as the secondary antibody and processed for immunofluorescence staining using the following primary antibodies: anti-mouse CD68 (1:200; Bio-Rad, catalog MCA1957) and Col1 (1:200; Abcam, catalog ab6308). Anti-rat and anti-rabbit Alexa Fluor 488–conjugated and Alexa Fluor 594–conjugated secondary antibodies, respectively, were used (1:400; Molecular Probes, Life Technologies). Images were acquired using an Olympus FV1000 confocal microscope and a Leica structural illumination DM 6000B microscope with Leica MMAF acquisition software.

### Flow cytometry and FACS.

Isolated cells were resuspended in 2% FBS/PBS solution and blocked with FcBlock on ice. Immune cell subpopulations were identified by staining for 20 minutes at room temperature with the following antibodies: Alexa Fluor 647–conjugated anti-CD45 (1:100; eBioscience; catalog 17-0112); FITC-conjugated anti-CD11b (1:100; eBioscience; catalog 17-0112); phycoerythrin-(PE)–conjugated anti-F4/80 (1:100; eBioscience; catalog 12-4801 or 53-4801); BV421–conjugated anti-Ly6G (1:100; BioLegend; catalog 141709); APC-conjugated anti-Ly6C (1:20; BioLegend; catalog 117326); BV421-conjugated anti-CD64 (1:100; BioLegend, catalog 139309); Alexa Fluor 700–conjugated anti-CD206 (1:100; BioLegend, catalog 141734); PE-Cy7–conjugated anti-MerTK (1:100; eBioscience, catalog 25-5751-82); BV421-conjugated anti-CD120a (1:100; R&D Systems, catalog FAB5538P); APC-Cy7–conjugated anti-Csf1r (1:100; BioLegend; catalog 135531, anti-Tmem173 (1:200; Abcam; catalog ab92605); and Alexa Fluor 488– (1:400; Invitrogen; catalog A21206) and APC-conjugated anti-CD120b (rat, 1:100; eBioscience; catalog 45-5932). 7AAD was added prior to cell analyses to exclude dead cells. Flow cytometric analyses and cell sorting were performed using a BD FACSAriaIII flow cytometer (BD Biosciences) and FlowJo software (Tree Star).

### Histological staining.

Injured hearts from mice were processed for paraffin embedding, cut into 10-μm sections on Superfrost Plus slides (Thermo Fisher Scientific), and deparaffinized using Histoclear (National Diagnostics). For picrosirius red staining, sections were stained using a Picro Sirius Red Stain Kit (Abcam) for 60 minutes according to the manufacturer’s protocol, and imaged on a Nikon TE2000 microscope under transmitted and polarized light. For Masson’s trichrome staining, sections were stained according to the manufacturer’s protocol (Trichrome Stain, Abcam, ab150686).

### Preparation of BMDMs.

BMDMs were prepared from the femurs and tibiae of 6- to 8-week-old *Rhbdf2^–/–^* and wild-type mice. Femurs and tibiae were isolated, ensuring that joints were kept intact, and placed in cold PBS. Muscle was removed using a sterile scalpel and scissors, the ends of the bones were cut in a ventilated hood, and bone marrow cells flushed out with cold PBS. Cells were filtered through a 40-μm cell strainer, centrifuged at 400 *g* for 10 minutes, and the supernatant was then removed. Cells were resuspended in RMPI 1640 medium (Sigma-Aldrich) containing 20% L929-conditioned medium, 10% FBS, 50 U/ml penicillin, 50 μg/ml streptomycin, and Glutamax. Cells were plated in 4 nontreated petri dishes per mouse, and allowed to differentiate for 7 days with additional differentiation medium added at day 4. On day 7, cells were detached using StemPro Accutase (Thermo Fisher Scientific) for 30 minutes to 1 hour at room temperature, centrifuged to pellet the cells, and either resuspended in normal nonconditioned media for subsequent plating or analyzed flow cytometry. Differentiation was assessed using the aforementioned flow cytometry panel, but replacing the associated fluorochrome with BV421-conjugated anti-MHCII (1:100 dilution; eBioscience; catalog 12-4801). Most of the produced cells were positive for CD68, CD14, and F4/80.

### Cardiac cine-MRI and analyses.

MRI was performed following MI as previously described (51). In brief, mice were anaesthetized with 2% isoflurane, and ECG electrodes inserted into the forepaws and a respiration loop was taped across the chest. The cradle containing the mice was lowered into an 11.7-T magnetic resonance system (Magnex Scientific). One-millimeter, short-axis stacks of cine-FLASH images were generated for the left ventricle. Long-axis 2-chamber and 4-chamber images were also acquired. Blinded image analysis was performed using ImageJ (NIH). All MRI parameters were calculated as previously described (53).

### Phagocytosis assay.

Phagocytosis was assessed by measuring the number of BMDMs that were able to take up latex beads coated with FITC-labeled rabbit IgG using a phagocytosis assay kit (FITC) (Cayman Chemical), according to the instruction manual. BMDMs from *Rhbdf2^–/–^* and wild-type mice were treated with the beads and cultured at 37°C for 1 hour. The uptake of beads into cells was captured using a microscope and quantified by the percentage of FITC-positive cells over total DAPI-positive cells.

### Western blotting.

BMDM cultures were lysed in RIPA buffer (50 mM Tris-HCl at pH 7.6, 150 mM NaCl, 1% NP-40, 0.5% deoxycholate, 0.1% SDS) supplemented with protease inhibitors (Protease Inhibitor Cocktail Tablet [Roche], 1 mM PMSF [Sigma-Aldrich] and 1 μg/ml aprotinin [Sigma-Aldrich]). The lysate was centrifuged at 13,000 *g* for 15 minutes at 4°C, and the supernatant recovered. Protein content was quantified, and 20 μg was run in an SDS-PAGE gel after incubation with Laemmli buffer/5% β-ME at 95°C for 5 minutes. Protein was transferred onto the membrane and primary antibodies for phospho-JNK (1:1,000 dilution; Cell Signaling Technologies, catalog 9251) and phospho-NF-κB (1:1,000 dilution, Cell Signaling Technologies, catalog 3033), and GAPDH (1:2,500 dilution, Millipore, catalog ab2302). All secondary antibodies were conjugated to HRP and imaged using enhanced chemiluminescence (GE Healthcare). Scanning densitometry was performed using ImageJ to quantify the relative protein levels across 3 biological replicates, and Student’s *t* test determined significance.

### Quantitative real-time PCR.

Total RNA was isolated from hearts using TRIzol Reagent (Thermo Fisher Scientific) and cDNA was synthesized using the Reverse Transcription System (Promega), both according to the manufacturer’s instructions. Quantitative real-time PCR was performed using SYBR Green on an ABI 7900 for the genes and primer sequences listed in Table 1. Expression levels were determined based on the 2^−ΔCT^ method.

### Statistics.

Statistical difference between groups was evaluated using Student’s *t* test (2-tailed) or 1-way ANOVA. A *P* value of 0.05 or less was considered statistically significant. All values and graphs present the mean value ± SEM.

### Study approval.

All animal experiments were carried out according to UK Home Office project license PPL 30/2987 compliant with the UK animals (Scientific Procedures) Act 1986 and approved by the local Biological Services Ethical Review Process.

## Author contributions

DNB, TJC, and PRR developed and designed the research studies. MF provided the iRhom2-mutant mice and reviewed the manuscript draft. DNB conducted most of the experiments and acquired data, with technical assistance from MGR and TJC. CAC performed MRI analyses and assisted with functional data interpretation. DNB and PRR interpreted data and primarily wrote and edited the manuscript, with input from all other authors.

## Supplementary Material

Supplemental data

## Figures and Tables

**Table 1:**
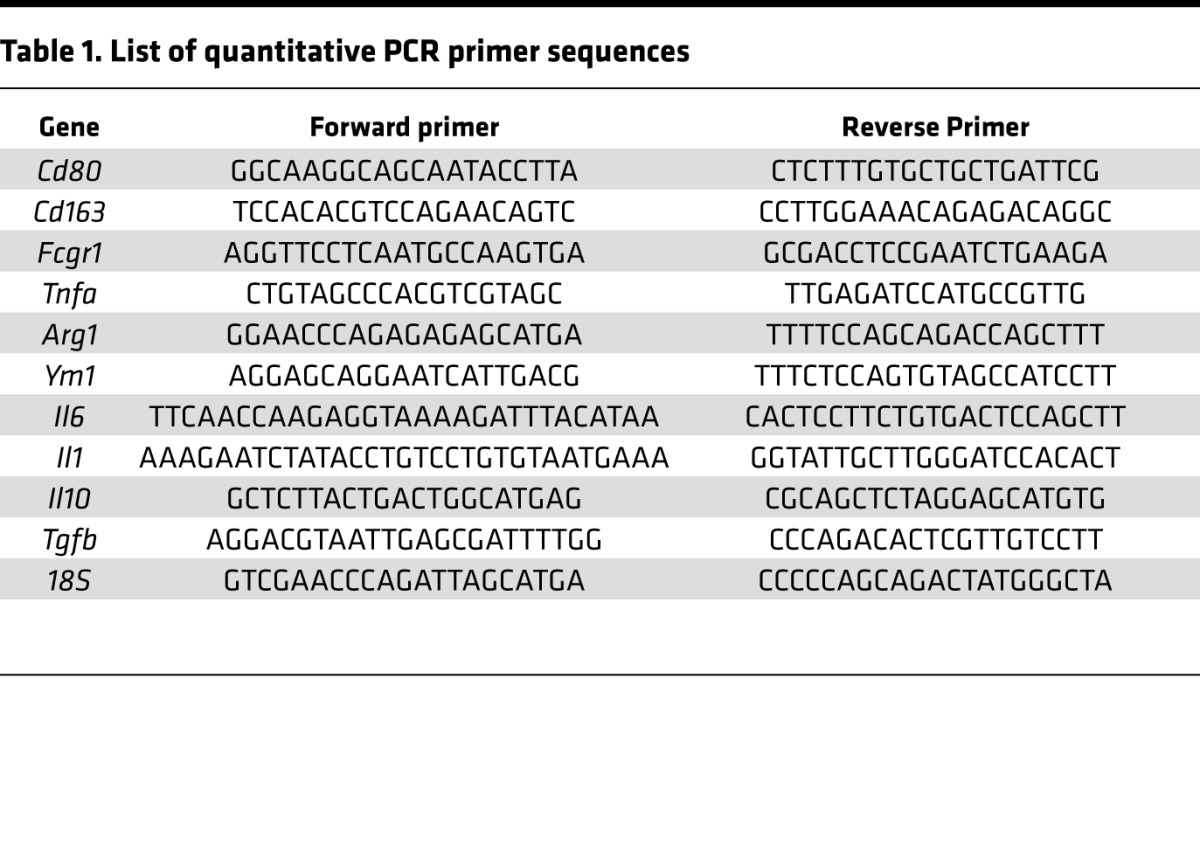
List of quantitative PCR primer sequences

## Version 1 02/08/2018

Electronic publication
