# Supplementary material for: iRhom2-mediated proinflammatory signalling regulates heart repair following myocardial infarction
Source: JCI Insight. 2018 Feb 8;3(3):e98268. doi: 10.1172/jci.insight.98268 (PMC5821194; doi:10.1172/jci.insight.98268)
Supplement: Supplemental data [file jciinsight-3-98268-s001.pdf]

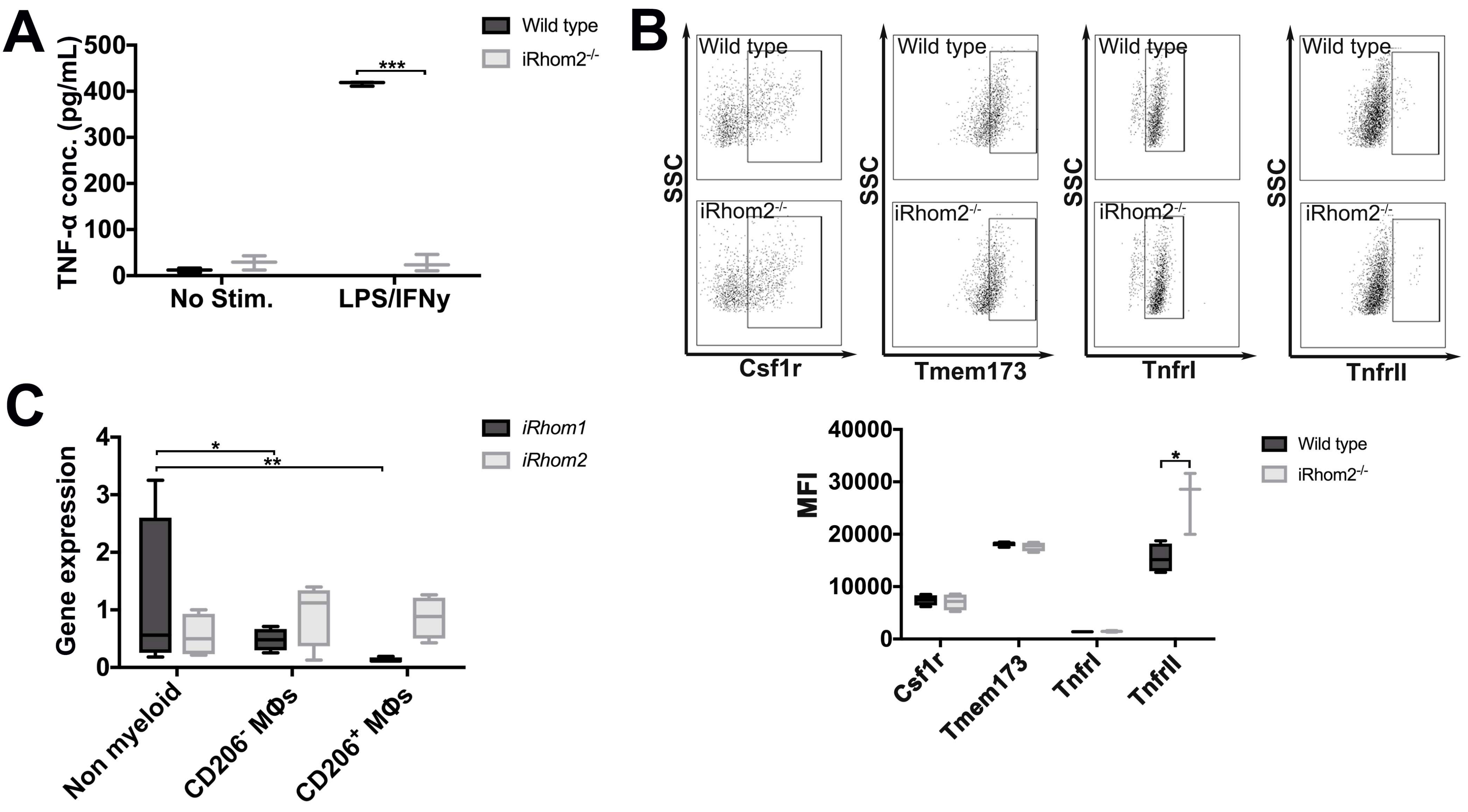

Barnette et al_Supplemental Figure 1

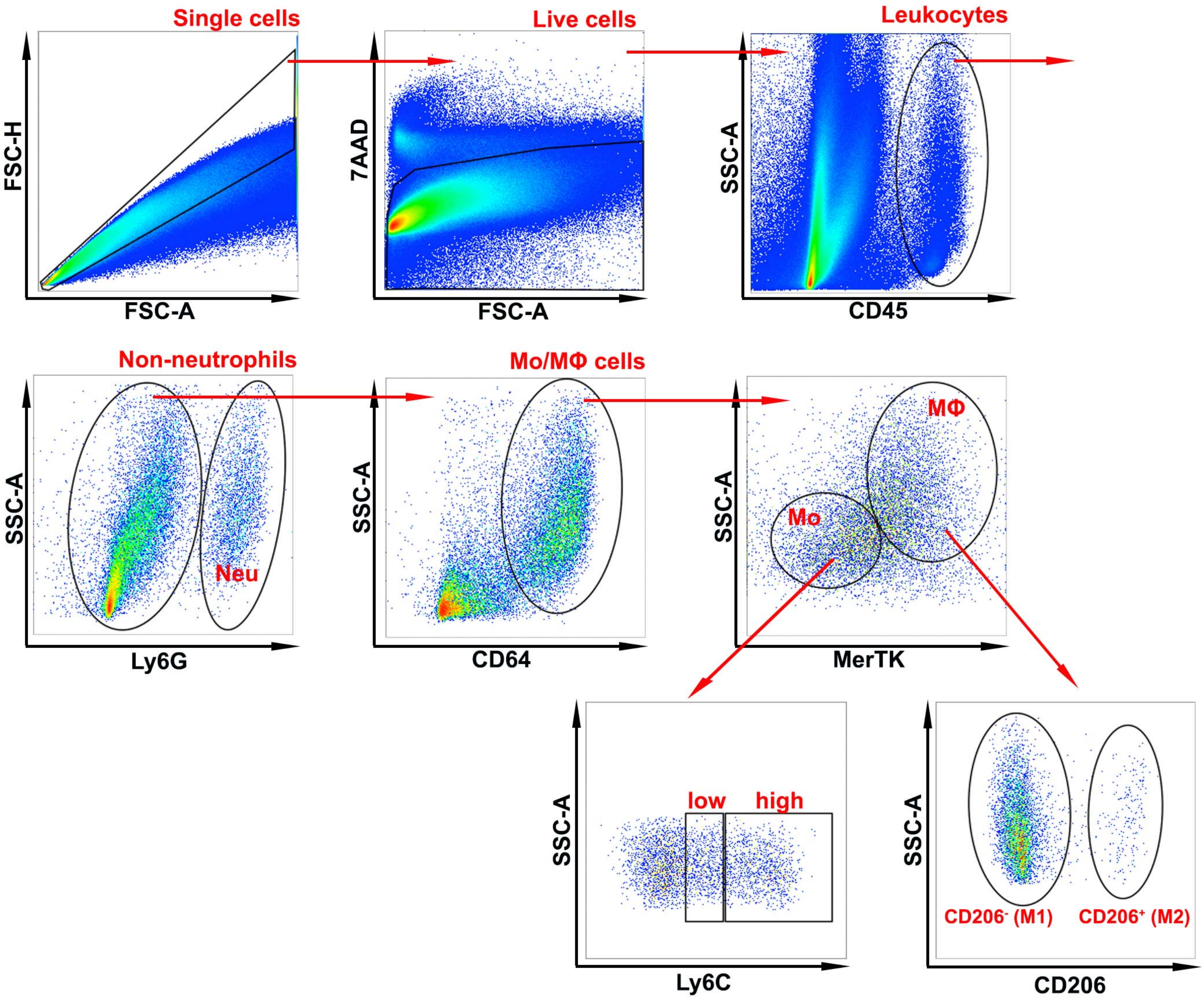

Barnette et al_Supplemental Figure 2

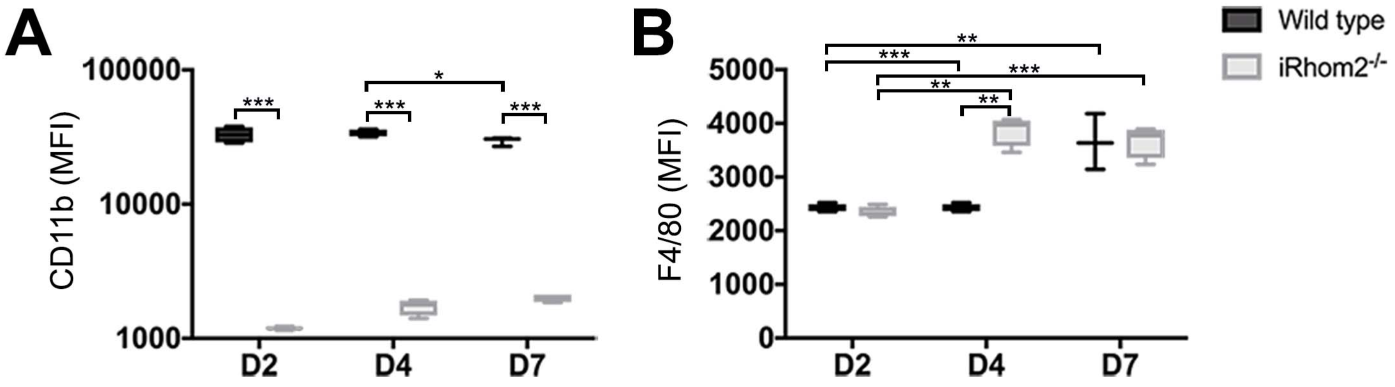

Barnette et al_Supplemental_Figure_ 3

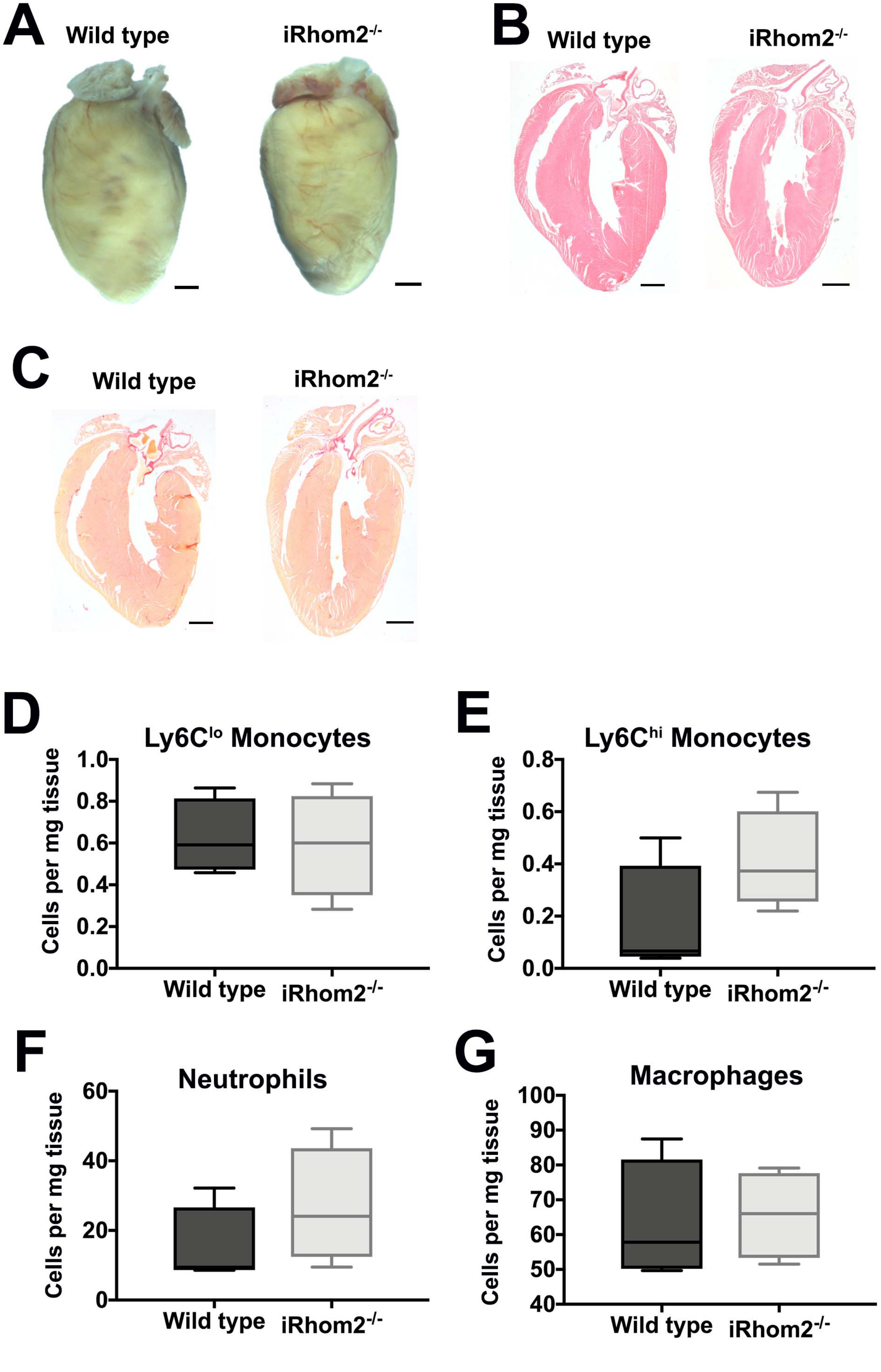

Barnette et al_Supplemental Figure 4

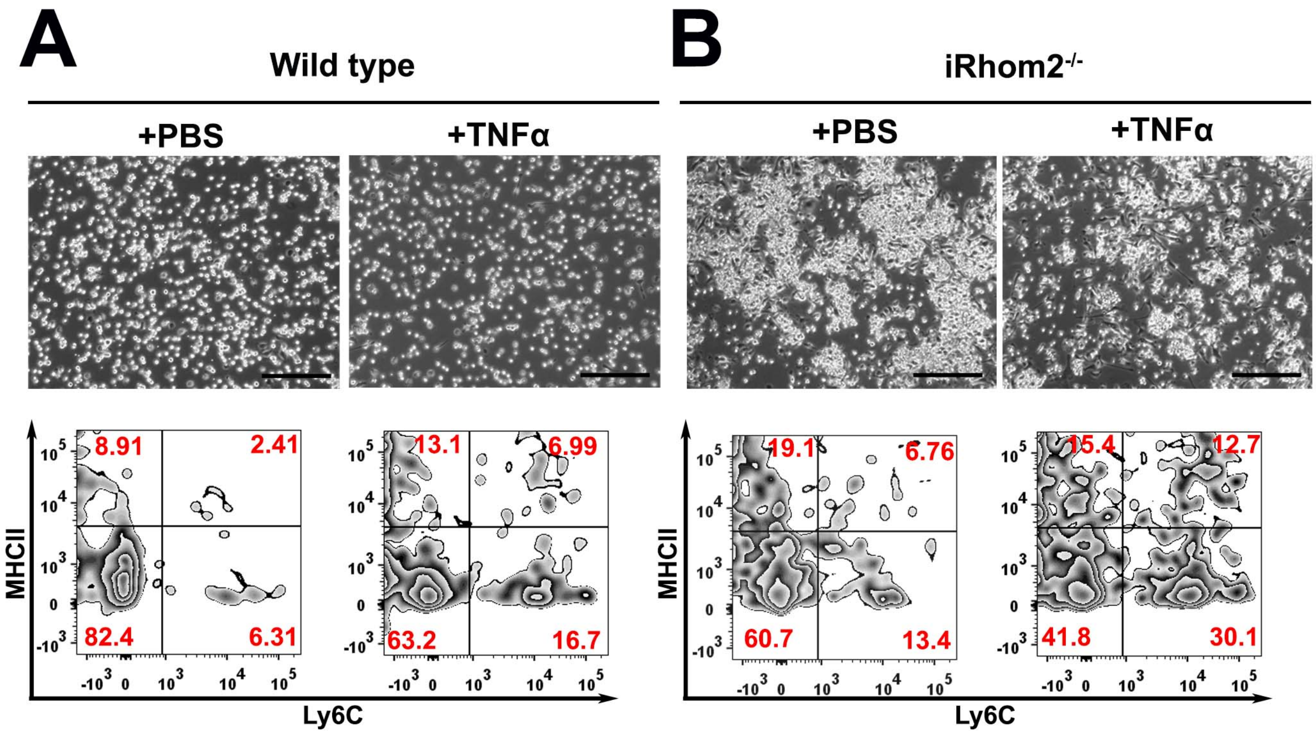

Barnette et al_Supplemental Figure 5
